# Association of Hematological Inflammatory Markers with T-MACS-Based Risk Stratification in Patients with Non-ST-Elevation Acute Coronary Syndrome

**DOI:** 10.3390/jcm15124399

**Published:** 2026-06-06

**Authors:** Ebru Çetin Kenan, Enad Kenan, Mehtap Bulut

**Affiliations:** 1 Department of Emergency Medicine, Bursa Yüksek İhtisas Training and Research Hospital, Bursa 16310, Türkiye; enadcanaan@gmail.com (E.K.); mbulut94@yahoo.com (M.B.); 2 Department of Emergency Medicine, Bursa City Hospital, Bursa 16110, Türkiye; 3 Department of Emergency Medicine, Bursa Orhangazi State Hospital, Bursa 16800, Türkiye

**Keywords:** acute coronary syndrome, NSTEMI, T-MACS score, hematological parameters, inflammatory markers, risk stratification, MACE

## Abstract

**Background:** Hematological parameters derived from complete blood count (CBC) are inexpensive and widely available markers with potential utility in risk stratification of acute coronary syndrome (ACS). However, their incremental prognostic value when used alongside contemporary risk stratification tools such as the Troponin-only Manchester Acute Coronary Syndrome (T-MACS) score remains unclear. **Methods:** In this prospective, single-center cohort study, 521 patients presenting with non-ST-segment elevation myocardial infarction (NSTEMI) or unstable angina were enrolled. Admission CBC parameters (white blood cell count, neutrophils, monocytes, red cell distribution width, mean platelet volume) and derived inflammatory indices (neutrophil-to-lymphocyte ratio, white blood cell-to-mean platelet volume ratio, lymphocyte-to-monocyte ratio, mean platelet volume-to-platelet ratio, and red cell distribution width-to-platelet ratio) were recorded. T-MACS risk scores were calculated, and patients were followed for 30-day major adverse cardiac events (MACE), mortality, and coronary interventions. Associations were assessed using univariate and multivariate logistic regression analyses. **Results:** Patients experiencing 30-day MACE or mortality had significantly higher white blood cell counts, neutrophil counts, and WMR values (all *p* < 0.05). Several hematological indices showed significant associations with T-MACS risk categories. In multivariate analysis, intermediate- and high-risk T-MACS classifications independently predicted 30-day MACE (OR 4.49, 95% CI:1.46–13.77, *p* = 0.009; OR 9.34, 95% CI:3.00–29.03, *p* < 0.001, respectively), whereas white blood cell count, neutrophil count, and WMR did not demonstrate independent prognostic value beyond T-MACS classification. **Conclusions:** Admission white blood cell count, neutrophil count, and WMR are associated with short-term adverse outcomes and T-MACS risk severity in patients with NSTE-ACS. However, these markers do not provide additional prognostic value beyond T-MACS classification. These findings suggest that CBC-derived inflammatory markers primarily reflect disease severity rather than incremental prognostic information in the contemporary high-sensitivity troponin era.

## 1. Introduction

Coronary artery disease (CAD) is characterized by narrowing or obstruction of the coronary arteries due to atherosclerotic plaque formation. Atherosclerosis is now widely recognized as a chronic inflammatory process [1,2]. Inflammation plays a central role in the development of acute coronary syndrome (ACS), particularly during plaque rupture and thrombus formation. Leukocytes and platelets contribute significantly to these mechanisms [1].

Several complete blood count (CBC)-derived parameters have been investigated as potential prognostic biomarkers in ACS. Previous studies demonstrated associations between white blood cell (WBC) count, neutrophil count, lymphocyte count, monocyte count, neutrophil-to-lymphocyte ratio (NLR), and adverse cardiovascular outcomes [1,3,4,5]. More recently, novel hematological indices such as the white blood cell-to-mean platelet volume ratio (WMR), lymphocyte-to-monocyte ratio (LMR), mean platelet volume-to-platelet ratio (MPR), and red cell distribution width-to-platelet ratio (RPR) have attracted increasing interest. However, evidence regarding their prognostic utility in ACS remains limited.

Early diagnosis and accurate risk stratification are essential in patients with non-ST-elevation acute coronary syndrome (NSTE-ACS). Several clinical risk scores, including HEART, TIMI, GRACE, and Troponin-only Manchester Acute Coronary Syndrome (T-MACS), have been developed to support clinical decision-making and estimate the risk of adverse cardiac events. In parallel with the widespread use of high-sensitivity cardiac troponin assays, contemporary management strategies increasingly emphasize rapid and reliable risk assessment in the emergency department [6].

Despite advances in risk stratification tools, there remains interest in simple, inexpensive, and widely available biomarkers that may provide additional prognostic information. To our knowledge, data regarding the relationship between T-MACS risk stratification and hematological inflammatory markers are limited.

Therefore, this study aimed to investigate the association between T-MACS risk categories and hematological inflammatory markers and to evaluate their predictive value for 30-day major adverse cardiac events (MACE) in patients presenting to the emergency department with unstable angina or non-ST-elevation myocardial infarction (NSTEMI).

## 2. Methods

### 2.1. Study Design and Setting

This prospective observational cohort study was conducted in the emergency department of Bursa Yuksek Ihtisas Training and Research Hospital between 1 June 2021 and 1 June 2022. The study protocol was approved by the institutional Ethics Committee (Approval No: 2011-KAEK-25 2021/05-22), and written informed consent was obtained from all participants.

### 2.2. Study Population

Adult patients aged ≥ 18 years presenting to the emergency department with suspected non-ST-elevation acute coronary syndrome (NSTE-ACS) were consecutively screened and included if diagnosed with non-ST-elevation myocardial infarction (NSTEMI) or unstable angina pectoris.

Patients were excluded if they had ST-segment elevation myocardial infarction (STEMI), pregnancy, active infection, malignancy, hematological or immunosuppressive disorders, severe hepatic or renal dysfunction, acute stroke, trauma-related chest pain, cardiac arrest on admission, prior coronary revascularization before presentation, or incomplete clinical data.

### 2.3. Data Collection

Demographic characteristics, cardiovascular risk factors, medical history, presenting symptoms, vital signs, electrocardiographic findings, laboratory results, and emergency department management data were recorded using a standardized case report form.

NSTE-ACS was diagnosed according to current guideline-based criteria, including ischemic symptoms, elevated cardiac troponin levels above the 99th percentile upper reference limit, dynamic ischemic electrocardiographic changes, or pathological Q waves consistent with myocardial ischemia.

### 2.4. Laboratory Measurements

Peripheral venous blood samples were obtained at emergency department admission prior to any therapeutic intervention.

Complete blood count (CBC) parameters were analyzed using a Mindray BC-6000 hematology analyzer (Mindray, Shenzhen, China). High-sensitivity cardiac troponin concentrations were measured using the ARCHITECT i2000SR system (Abbott Laboratories, Chicago, IL, USA).

CBC parameters included white blood cell (WBC) count, neutrophil count, lymphocyte count, monocyte count, hemoglobin, hematocrit, platelet count, red cell distribution width (RDW), platelet distribution width (PDW), and mean platelet volume (MPV).

The following hematological inflammatory indices were calculated:

- Neutrophil-to-lymphocyte ratio (NLR) = neutrophils/lymphocytes
- White blood cell-to-mean platelet volume ratio (WMR) = WBC/MPV
- Lymphocyte-to-monocyte ratio (LMR) = lymphocytes/monocytes
- Mean platelet volume-to-platelet ratio (MPR) = MPV/platelet count
- Red cell distribution width-to-platelet ratio (RPR) = RDW/platelet count

### 2.5. T-MACS Risk Stratification

Troponin-only Manchester Acute Coronary Syndrome (T-MACS) probability scores were calculated using the validated original model described by Body et al. [7]. The model integrates clinical variables, electrocardiographic findings, and high-sensitivity cardiac troponin values at presentation.

Patients were classified into four predefined risk categories: very low, low, moderate, and high risk according to estimated probability of acute coronary syndrome.

### 2.6. Outcome Measures

Patients were followed for 30 days after index presentation. The primary endpoint was 30-day major adverse cardiac events (MACE), defined as a composite of all-cause mortality, myocardial infarction, or coronary revascularization (percutaneous coronary intervention or coronary artery bypass grafting).

Secondary outcomes included 30-day all-cause mortality and percutaneous coronary intervention.

### 2.7. Statistical Analysis

Statistical analyses were performed using IBM SPSS Statistics for Windows, version 28.0. (IBM Corp., Armonk, NY, USA). Continuous variables were assessed for normality using the Kolmogorov–Smirnov test and visual inspection of distributions.

Continuous variables were expressed as mean ± standard deviation or median (interquartile range), as appropriate. Categorical variables were presented as frequencies and percentages.

Comparisons between two independent groups were performed using Student’s *t*-test or Mann–Whitney U test. Comparisons among multiple groups were performed using one-way ANOVA or Kruskal–Wallis test, as appropriate. Categorical variables were analyzed using the chi-square test or Fisher’s exact test.

Variables showing significant associations in univariate analyses were entered into multivariate logistic regression models to identify independent predictors of 30-day MACE. Odds ratios (ORs) with 95% confidence intervals (CIs) were reported. For multivariable model development, the overall cohort was randomly divided into training (60%) and validation (40%) subsets based on the methodology described by Sung et al. Multivariable logistic regression analyses were performed in the training cohort (n = 313), while the remaining patients constituted the validation cohort [8].

Model calibration was assessed using the Hosmer–Lemeshow goodness-of-fit test. A two-sided *p* value < 0.05 was considered statistically significant.

No formal sample size calculation was performed. All consecutive eligible patients presenting during the study period were included.

## 3. Results

During the study period, 735 patients presented with suspected acute coronary syndrome. Of these, 214 patients with ST-segment elevation myocardial infarction and patients with alternative non-cardiac diagnoses were excluded. A total of 521 patients with confirmed NSTE-ACS (NSTEMI or unstable angina) were ultimately included in the final analysis.

The median age was 59 years (19–96), and 371 (71.2%) were male. Typical chest pain was reported in 233 patients (44.7%), sweating in 250 (48.0%), and right arm or shoulder pain in 209 (40.1%). The median time from symptom onset to emergency department presentation was 360 min (IQR 120–2880), indicating substantial variability in presentation timing among patients. Cardiovascular risk factors were present in 459 patients (88.1%), most commonly hypertension (55.9%) and diabetes mellitus (34.9%). NSTEMI accounted for 293 cases (56.2%), and 454 patients (87.1%) were hospitalized.

According to Troponin-only Manchester Acute Coronary Syndrome classification, 240 patients (46.1%) were in the high-risk group, 212 (40.7%) in the moderate-risk group, 28 (5.4%) in the low-risk group, and 41 (7.9%) in the very low-risk group.

Within 30 days, major adverse cardiac events (MACE) occurred in 263 patients (50.3%), while mortality occurred in 19 patients (3.6%). Percutaneous coronary intervention was performed in 222 patients (42.6%), coronary artery bypass grafting in 33 (6.3%), and myocardial infarction occurred in 35 patients (6.7%) (Table 1).

Admission hematological parameters and troponin levels showed significant differences between outcome groups. Patients with MACE had higher white blood cell (WBC) counts, neutrophil counts, and white blood cell-to-mean platelet volume ratio (WMR) compared with those without MACE. Troponin levels were also significantly higher in the MACE group, reflecting a greater degree of myocardial injury.

Similar patterns were observed for 30-day mortality, with non-survivors demonstrating higher WBC counts, neutrophil counts, WMR values, and troponin levels. Among patients undergoing percutaneous coronary intervention, WBC count, neutrophil count, WMR, and troponin were also significantly elevated.

In contrast, neutrophil-to-lymphocyte ratio (NLR), lymphocyte-to-monocyte ratio (LMR), mean platelet volume (MPV), and red cell distribution width (RDW) did not show statistically significant differences across outcome groups.

Overall, WBC count, neutrophil count, WMR, and troponin appeared to be the most consistently associated parameters with short-term adverse outcomes in this cohort (Table 2).

T-MACS risk classification was significantly associated with 30-day MACE (*p* < 0.01). Among patients with MACE, 59.7% were classified as high risk, whereas only 2.7% were in the very low-risk group. In contrast, among patients without MACE, 32.2% were high risk and 13.2% were very low risk (Table 3).

Significant differences were observed in WBC count, neutrophil count, monocyte count, RDW, LMR, NLR, WMR, and troponin levels across T-MACS risk categories (*p* < 0.05 for all comparisons) (Table 4). Hematological and inflammatory markers demonstrated a stepwise increase with higher T-MACS risk classification.

**Figure 1:**
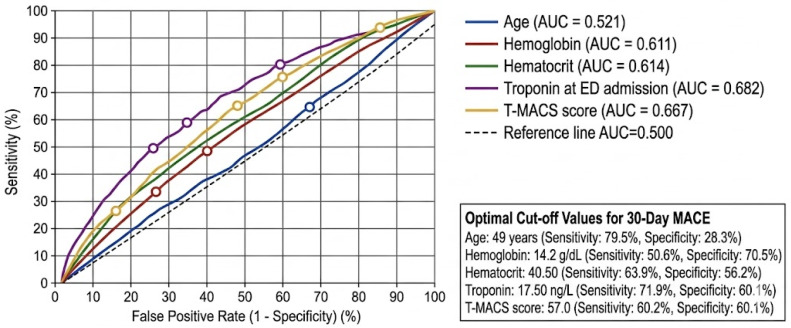
ROC Curve Analysis of Age, Hemoglobin, Hematocrit, Troponin at ED Admission, and T-MACS Score for the Prediction of 30-Day MACE.

Receiver operating characteristic (ROC) curve analysis was performed to evaluate the diagnostic performance of age, hemoglobin, hematocrit, troponin level at emergency department (ED) admission, and T-MACS score for predicting 30-day major adverse cardiovascular events (MACE). Among the evaluated variables, troponin level at admission demonstrated the highest discriminative ability (AUC = 0.682; 95% CI:0.640–0.722; *p* < 0.001), followed by the T-MACS score (AUC = 0.667; 95% CI:0.624–0.707; *p* < 0.001). Hemoglobin (AUC = 0.611; 95% CI:0.568–0.654; *p* < 0.001) and hematocrit (AUC = 0.614; 95% CI:0.570–0.656; *p* < 0.001) showed moderate predictive performance, whereas age had no significant discriminative value (AUC = 0.521; 95% CI:0.477–0.565; *p* = 0.406). Optimal cut-off values, sensitivity, and specificity for each variable are presented in Figure 1.

**Figure 2:**
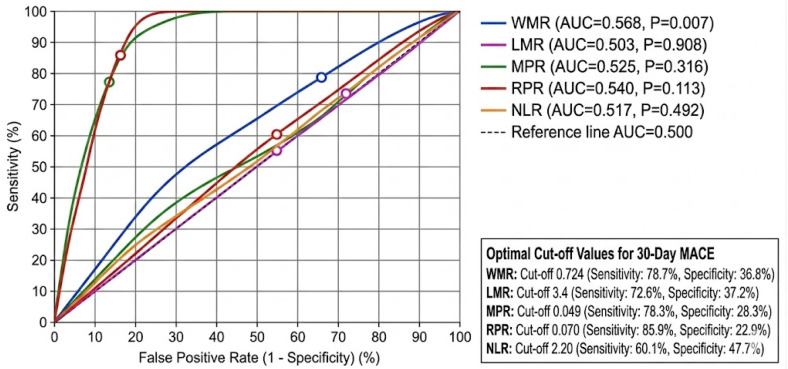
ROC Analysis of Select Biomarker Ratios for Predicting 30-Day MACE. AUC: Area under the curve; CI: Confidence interval; NLR: neutrophil-to-lymphocyte ratio; WMR: WBC/MPV ratio; LMR: lymphocyte-to-monocyte ratio; MPR: mean platelet volume/platelet ratio; RPR: red cell distribution width/platelet ratio; MACE: major adverse cardiovascular events.

Receiver operating characteristic (ROC) curve analysis was performed to evaluate the diagnostic performance of neutrophil-to-lymphocyte ratio (NLR), white blood cell/mean platelet volume ratio (WMR), lymphocyte-to-monocyte ratio (LMR), mean platelet volume/platelet ratio (MPR), and red cell distribution width/platelet ratio (RPR) for predicting 30-day major adverse cardiovascular events (MACE). Among all evaluated hematological ratios, only WMR demonstrated a statistically significant association with 30-day MACE (*p* = 0.007). The WMR showed a modest discriminative ability with an AUC of 0.568 (95% CI:0.524–0.611; *p* = 0.007). The optimal cut-off value for WMR was 0.724, yielding a sensitivity of 78.7% and a specificity of 36.8%. LMR (AUC = 0.503; 95% CI:0.459–0.547; *p* = 0.907), MPR (AUC = 0.525; 95% CI:0.481–0.569; *p* = 0.316), RPR (AUC = 0.540; 95% CI:0.496–0.583; *p* = 0.113), and NLR (AUC = 0.517; 95% CI:0.474–0.561; *p* = 0.492) did not demonstrate statistically significant predictive value for 30-day MACE. Optimal cut-off values with corresponding sensitivity and specificity for each parameter are presented in Figure 2.

**Figure 3:**
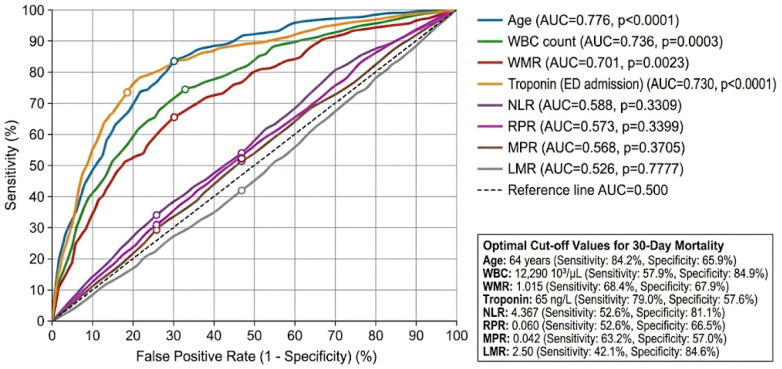
ROC Analysis of Clinical and Biomarker Variables for Predicting 30-Day Mortality.

ROC analysis was performed to evaluate the diagnostic value of age, WBC, NLR, WMR, MPR, RPR, LMR, and admission troponin levels for 30-day mortality. The analysis demonstrated that age, WBC, WMR, and troponin were significantly associated with 30-day mortality (*p* < 0.001, *p* = 0.0003, *p* = 0.0023, and *p* < 0.0001, respectively) (Figure 3).

For 30-day mortality prediction, the AUC for age was 0.776 (95% CI:0.738–0.811, *p* < 0.001). The optimal cutoff value for age was 64 years, with a sensitivity of 84.2% and a specificity of 65.9%. The AUC for WBC was 0.736 (95% CI:0.696–0.773, *p* = 0.0003), and the optimal cutoff value was 12.290 × 10^3^/μL, yielding a sensitivity of 57.9% and a specificity of 84.9% (Figure 3).

The AUC for NLR was 0.588 (95% CI:0.544–0.630, *p* = 0.3309). The optimal cutoff value for NLR was 4.367, with a sensitivity of 52.6% and a specificity of 81.1%. The AUC for WMR was 0.701 (95% CI:0.660–0.740, *p* = 0.0023), and the optimal cutoff value was 1.015, with a sensitivity of 68.4% and a specificity of 67.9% (Figure 3).

The AUC for MPR was 0.568 (95% CI:0.525–0.611, *p* = 0.3705), and the optimal cutoff value was 0.042, with a sensitivity of 63.2% and a specificity of 57.0%. The AUC for RPR was 0.573 (95% CI:0.529–0.616, *p* = 0.3399), and the optimal cutoff value was 0.060, with a sensitivity of 52.6% and a specificity of 66.5%. The AUC for LMR was 0.526 (95% CI:0.483–0.570, *p* = 0.7777), and the optimal cutoff value was 2.50, with a sensitivity of 42.1% and a specificity of 84.6% (Figure 3).

For admission troponin levels, the AUC was 0.730 (95% CI:0.690–0.768, *p* < 0.0001). The optimal cutoff value was 65 ng/L, with a sensitivity of 79.0% and a specificity of 57.6% (Figure 3).

**Figure 4:**
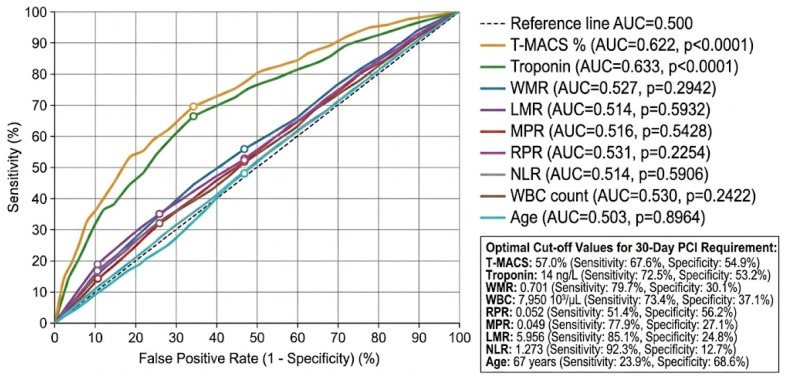
ROC Curve Analysis of Clinical, Biomarker, and Score-based Variables for Predicting 30-Day PCI Requirement.

ROC analysis was performed to evaluate the diagnostic performance of age, WBC, NLR, WMR, MPR, RPR, LMR, admission troponin levels, and T-MACS percentage for 30-day percutaneous coronary intervention (PCI) requirement. The AUC for age was 0.503 [95% CI:0.460–0.547, (*p* = 0.8964)], and at a cut-off value of 67 years, the sensitivity and specificity were 23.9% and 68.6%, respectively. The AUC for WBC was 0.530 [95% CI:0.486–0.573, (*p* = 0.2422)], with a cut-off value of 7.950 × 10^3^/μL yielding a sensitivity of 73.4% and a specificity of 37.1%. The AUC for WMR (WBC/MPV ratio) was 0.527 [95% CI:0.483–0.570, (*p* = 0.2942)], and at a cut-off value of 0.701, sensitivity and specificity were 79.7% and 30.1%, respectively. The AUC for NLR (neutrophil-to-lymphocyte ratio) was 0.514 [95% CI:0.470–0.557, (*p* = 0.5906)], with a cut-off value of 1.273 showing sensitivity of 92.3% and specificity of 12.7%. The AUC for MPR (MPV/platelet ratio) was 0.516 [95% CI:0.472–0.559, (*p* = 0.5428)], and at a cut-off value of 0.049, sensitivity and specificity were 77.9% and 27.1%, respectively. The AUC for RPR (RDW/platelet ratio) was 0.531 [95% CI:0.487–0.575, (*p* = 0.2254)], with a cut-off value of 0.052 yielding sensitivity of 51.4% and specificity of 56.2%. The AUC for LMR (lymphocyte-to-monocyte ratio) was 0.514 [95% CI:0.470–0.557, (*p* = 0.5932)], and at a cut-off value of 5.956, sensitivity and specificity were 85.1% and 24.8%, respectively. For admission troponin levels, the AUC was 0.633 [95% CI:0.590–0.674, (*p* < 0.0001)], with a cut-off value of 14 ng/L yielding sensitivity of 72.5% and specificity of 53.2%. The AUC for T-MACS percentage was 0.622 [95% CI:0.579–0.664, (*p* < 0.0001)], and at a cut-off value of 57.0%, sensitivity and specificity were 67.6% and 54.9%, respectively (Figure 4).

**Figure 5:**
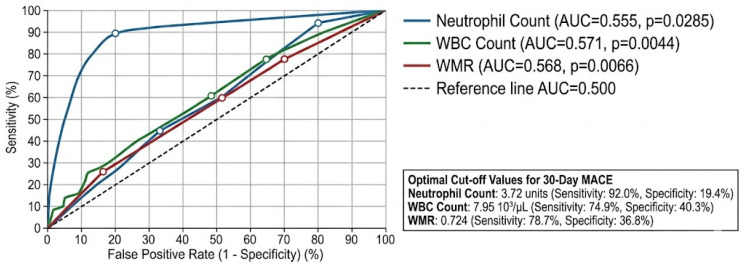
ROC Analysis of Neutrophil Count, WBC Count, and WMR for Predicting 30-Day MACE.

ROC analysis was performed to evaluate the diagnostic performance of neutrophil count, WBC, and WMR for 30-day major adverse cardiovascular events (MACE). In this analysis, the AUC for neutrophil count was 0.555 [95% CI:0.511–0.598, (*p* = 0.0285)], and at a cut-off value of 3.72 × 10^3^/µL, the sensitivity and specificity were 92.0% and 19.4%, respectively. The AUC for WBC was 0.571 [95% CI:0.528–0.614, (*p* = 0.0044)], with a cut-off value of 7.95 × 10^3^/μL yielding a sensitivity of 74.9% and a specificity of 40.3%. The AUC for WMR (WBC/MPV ratio) was 0.568 [95% CI:0.524–0.611, (*p* = 0.0066)], and at a cut-off value of 0.724, the sensitivity and specificity were 78.7% and 36.8%, respectively (Figure 5).

Variables with statistically significant ROC performance were dichotomized according to ROC-derived optimal cut-off values before inclusion in the multivariable regression model.

To construct the multivariable prediction model, the study cohort was randomly divided into training and validation subsets using a 6:4 ratio according to the previously described methodology. The training cohort included 313 patients and was used for multivariable logistic regression analysis.

The multivariate logistic regression model was statistically significant (χ^2^ = 43.92, *p* < 0.001) and demonstrated good calibration (Hosmer–Lemeshow test *p* = 0.715).

Compared with the very low-risk group, moderate-risk patients had a significantly increased risk of 30-day MACE (OR 4.49; 95% CI:1.46–13.77; *p* = 0.009), and high-risk patients had an even higher risk (OR 9.34; 95% CI:3.00–29.03; *p* < 0.001).

However, after adjustment, elevated WBC, WMR, and neutrophil levels were not independently associated with 30-day MACE and did not significantly improve risk prediction when added to the T-MACS model (Table 5).

## 4. Discussion

Recent studies have evaluated various complete blood count (CBC) components and CBC-derived inflammatory indices as potential prognostic markers in patients with acute coronary syndrome (ACS) [1,9,10]. In the present study, we investigated the relationship between hematological inflammatory markers, T-MACS risk classification, and 30-day outcomes in patients with non-ST-elevation acute coronary syndrome (NSTE-ACS). Our findings demonstrated that WBC count, neutrophil count, and WMR were significantly associated with 30-day MACE, mortality, and PCI in univariate analyses. In addition, several inflammatory markers showed a stepwise increase across higher T-MACS risk categories. However, these parameters did not retain independent prognostic significance after adjustment for T-MACS classification in multivariate analysis.

The median age of our cohort was comparable to previously published NSTEMI populations. Dehghani et al. reported no significant age difference between MACE-positive and MACE-negative NSTEMI patients in a cohort of 490 patients [1], whereas Wang et al. observed older age among patients who developed MACE [11]. Similarly, age was not significantly associated with 30-day MACE in our study. These differences across studies may reflect variations in patient characteristics, comorbidity burden, and inclusion criteria.

The incidence of 30-day MACE in our cohort was higher than that reported in previous ACS studies [1,9,12]. While prior investigations reported MACE rates ranging from 16.5% to 23.2%, 50.3% of our patients experienced MACE within 30 days. This finding may be explained by the tertiary referral nature of our center, where patients with more complex clinical presentations and higher baseline cardiovascular risk are more frequently managed. Importantly, the high MACE rate was largely driven by inclusion of coronary revascularization procedures within the composite endpoint. In addition, inclusion of coronary revascularization in the composite endpoint may have contributed to the higher observed event rate. Because coronary revascularization procedures may partially reflect physician decision-making and institutional treatment strategies rather than spontaneous adverse clinical deterioration, inclusion of PCI and CABG within the composite endpoint may have introduced treatment-related bias into MACE estimation. Therefore, the observed associations should be interpreted with caution.

Inflammation plays a central role in plaque destabilization, endothelial dysfunction, and thrombus formation during ACS. Leukocyte activation contributes to cytokine release, oxidative stress, and vascular injury, which may explain the relationship between inflammatory hematological markers and adverse cardiovascular outcomes. In our study, elevated WBC count was associated with both 30-day MACE and mortality. Similar findings were previously reported by Babes et al., who demonstrated a significant association between elevated WBC levels and adverse outcomes in ACS patients [13]. Dehghani et al. also observed significantly higher WBC counts among NSTEMI patients who developed MACE [1]. Our findings further support the role of systemic inflammatory activation in short-term prognosis among patients with NSTE-ACS.

Neutrophil count was also significantly associated with adverse outcomes in our cohort. Patients who developed MACE or mortality had higher neutrophil counts compared with those without events. Neutrophils are known to contribute to atherosclerotic plaque instability and microvascular injury through release of proteolytic enzymes and reactive oxygen species. Previous studies by Karaoğlu et al., Dehghani et al., and Wang et al. similarly demonstrated an association between elevated neutrophil counts and MACE in ACS populations [1,11,12]. The consistency of these findings across different cohorts supports the potential value of neutrophil-mediated inflammatory activity as a marker of disease severity in ACS.

Among CBC-derived inflammatory indices, WMR demonstrated one of the strongest associations with adverse outcomes in our study. Patients with elevated WMR values had significantly higher rates of MACE, mortality, and PCI. WMR may reflect both inflammatory burden and platelet activation, which are central mechanisms in ACS pathophysiology. Previous studies by Demir et al., Ommen et al., Karaoğlu et al., and Dehghani et al. similarly reported higher WMR values in ACS patients with adverse cardiovascular outcomes [1,12,14,15]. Our findings are consistent with this growing body of evidence suggesting that WMR may serve as a readily available marker of short-term cardiovascular risk.

In contrast, NLR, LMR, MPV, RDW, and MPR were not significantly associated with 30-day MACE in our cohort. Although several previous studies demonstrated prognostic associations for these parameters in ACS populations [4,10,12,16,17,18,19,20,21,22,23,24,25,26], results across the literature remain inconsistent. Differences in study design, ACS subtype distribution, timing of blood sampling, endpoint definitions, and patient selection may partially explain these discrepancies. Importantly, many prior studies focused predominantly on STEMI populations or critically ill patients, whereas our study specifically evaluated a contemporary NSTE-ACS cohort managed in the high-sensitivity troponin era.

Our analysis also demonstrated significant relationships between T-MACS risk categories and multiple inflammatory markers, including WBC, neutrophil count, monocyte count, RDW, LMR, NLR, WMR, and troponin levels. Hematological parameters generally increased in parallel with higher T-MACS risk classification. These findings suggest that inflammatory activation may reflect the overall clinical severity captured by established risk stratification systems.

However, despite significant univariate associations, WBC count, neutrophil count, and WMR did not remain independent predictors of 30-day MACE after adjustment for T-MACS classification. In contrast, moderate- and high-risk T-MACS categories remained strongly associated with adverse outcomes. These findings suggest that although inflammatory hematological markers reflect disease activity and clinical instability, their incremental prognostic contribution beyond contemporary clinical decision tools may be limited.

In the contemporary era of high-sensitivity cardiac troponin assays and accelerated diagnostic pathways, the additive value of novel biomarkers remains uncertain. Established risk stratification tools such as T-MACS already incorporate major determinants of myocardial injury and clinical risk. Therefore, inflammatory hematological markers may partially overlap with prognostic information already captured by troponin elevation, electrocardiographic abnormalities, and clinical presentation. This overlap may explain why several CBC-derived markers lose independent significance after multivariate adjustment.

Our findings should be interpreted in light of several limitations. First, this was a single-center study conducted at a tertiary referral hospital, which may limit generalizability. Second, the sample size was relatively modest, particularly for mortality analyses. Third, only admission hematological parameters were evaluated, and serial measurements were not available. Fourth, follow-up duration was limited to 30 days. Finally, external validation of our findings in independent cohorts was not performed.

Overall, our results suggest that routinely available hematological inflammatory markers are associated with short-term adverse outcomes and increasing T-MACS risk severity in patients with NSTE-ACS. However, these markers did not provide substantial independent prognostic value beyond T-MACS classification in multivariate analysis. Further large-scale prospective studies are needed to determine whether selected inflammatory biomarkers may improve contemporary risk stratification strategies in ACS.

## 5. Conclusions

Our study showed that readily available hematological parameters, particularly WBC count, neutrophil count, and WMR, were associated with short-term adverse outcomes in patients with NSTE-ACS. Inflammatory markers also demonstrated significant variation across T-MACS risk categories, supporting the relationship between systemic inflammatory activity and clinical risk severity.

However, these hematological parameters did not provide meaningful independent prognostic value after adjustment for T-MACS classification. This finding suggests that CBC-derived inflammatory markers may reflect disease burden and clinical instability rather than contribute substantial additional predictive information beyond established risk stratification tools.

In the era of high-sensitivity troponin-based diagnostic pathways, T-MACS appears to remain a robust predictor of short-term outcomes in patients with NSTE-ACS. Hematological parameters may still serve as supportive indicators of inflammatory status, but their role as independent prognostic tools remains limited. Further multicenter prospective studies are needed to determine whether novel biomarkers can improve risk prediction beyond contemporary clinical decision models.

## Figures and Tables

**Table 1:** Clinical and Demographic Information Table.

Age (Years) *		59 (19–96)
Gender	Male	371 (71.2%)
	Female	150 (28.8%)
Pain Type ^#^	Typical	233 (44.7%)
	Atypical	64 (12.3%)
	Typical + Atypical	224 (43.0%)
Symptoms ^#^	Sweating	250 (48.0%)
	Right Arm/Shoulder Pain	209 (40.1%)
	Nausea	74 (14.2%)
	Vomiting	74 (14.2%)
	Syncope/Presyncope	27 (5.2%)
	Crescendo	80 (15.4%)
Pain Onset Time (min) *		360 (IQR 120–2880)
Risk Factor ^#^		459 (88.1%)
Risk Factors ^#^	Hypertension	291 (55.9%)
	Diabetes Mellitus	182 (34.9%)
	Obesity	45 (8.6%)
	Smoking	172 (33.0%)
	Family History	59 (11.3%)
	Hyperlipidemia	71 (13.6%)
	Previous Myocardial Infarction	249 (47.8%)
	Previous Coronary Bypass Graft	55 (10.6%)
	Previous Cerebrovascular Disease	13 (2.5%)
	Peripheral Artery Disease	8 (1.5%)
Emergency Department Diagnosis ^#^	Unstable Angina Pectoris	228 (43.7%)
	Non-ST Elevation Myocardial Infarction	293 (56.2%)
Hospitalization ^#^		454 (87.1%)
T-MACS Score ^#^	Very Low Risk	41 (7.9%)
	Low Risk	28 (5.4%)
	Moderate Risk	212 (40.7%)
	High Risk	240 (46.1%)
MACE in the First 30 Days ^#^		263 (50.3%)
Mortality in the First 30 Days ^#^		19 (3.6%)
STEMI in the First 30 Days ^#^		35 (6.7%)
Percutaneous Coronary Intervention in the First 30 Days ^#^		222 (42.6%)
Coronary Artery Bypass Graft in the First 30 Days ^#^		33 (6.3%)
Total ^#^		521

* Data are presented as median (range) unless otherwise indicated. ^#^ Data are presented as n (%).

**Table 2:** Comparison of hematological parameters, inflammatory indices, and troponin levels according to 30-day clinical outcomes (MACE, mortality, and PCI).

Variable	30-Day MACE (+)	30-Day MACE (−)	*p* Value	30-Day Mortality (+)	30-Day Mortality (−)	*p* Value	PCI (+)	PCI (−)	*p* Value
WBC (×10^3^/µL)	9.45	8.77	0.005	12.38	9.05	<0.001	9.37	8.92	<0.001
Neutrophil (×10^3^/µL)	5.80	5.41	0.029	8.00	5.64	0.018	5.72	5.61	0.018
WMR (ratio)	0.90	0.84	0.007	1.08	0.87	0.003	0.87	0.87	0.003
NLR (ratio)	2.49	2.36	0.492	4.37	2.42	0.195	2.47	2.38	0.195
LMR (ratio)	4.27	4.24	0.907	3.79	4.26	0.696	4.24	4.31	0.696
MPV (fL)	10.5	10.6	0.429	11.0	10.5	0.061	10.5	10.6	0.061
RDW (%)	13.6	13.7	0.275	14.1	13.6	0.198	13.6	13.7	0.198
Troponin (ng/L)	106.3	8.45	<0.001	638.1	31.15	0.001	87.9	12.0	0.001

Data are presented as median values.

**Table 3:** T-MACS Risk Class MACE (+) 30 Days.

Association Between T-MACS Risk Classification and 30-Day MACE					
			MACE (+) 30 Days		Total
			No	Yes	
T-MACS RISK CLASS	VERY LOW RISK	MACE (+) (n)	34	7	41
		MACE (+) (%)	13.2%	2.7%	7.9%
	HIGH RISK	MACE (+) (n)	83	157	240
		MACE (+) (%)	32.2%	59.7%	46.1%
	LOW RISK	MACE (+) (n)	22	6	28
		MACE (+) (%)	8.5%	2.3%	5.4%
	MODERATE RISK	MACE (+) (n)	119	93	212
		MACE (+) (%)	46.1%	35.4%	40.7%
Total		MACE (+) (n)	258	263	521

**Table 4:** Data are presented as median values. Comparisons across T-MACS risk categories were performed using the Kruskal–Wallis test.

T-MACS RISK CLASS		WBC (×10^3^/µL)	Neutrophil (×10^3^/µL)	Monocyte (×10^3^/µL)	RDW (%)	LMR (Ratio)	NLR (Ratio)	WMR (Ratio)	Troponin (ng/L)
VERY LOW RISK	n	41	41	41	41	41	41	41	41
	Median	7.84	4.59	0.51	13.3	4.57	1.94	0.76	0.8
LOW RISK	n	28	28	28	28	28	28	28	28
	Median	7.39	4.6	0.50	13.65	4.92	1.79	0.72	1.8
MODERATE RISK	n	212	212	212	212	212	212	212	212
	Median	8.77	5.31	0.55	13.6	4.57	2.15	0.82	6
HIGH RISK	n	240	240	240	240	240	240	240	240
	Median	9.99	6.56	0.61	13.8	3.71	2.95	0.95	501.35
*p* value		<0.001	<0.001	0.006	0.025	<0.001	<0.001	<0.001	<0.001

**Table 5:** Multivariable logistic regression analysis for 30-day MACE in the training cohort (n = 313).

n = 313	Multivariate Logistic Regression Model	
	OR (%95CI)	*p*
T-MACS Classification		
VERY LOW RISK	1	-
LOW RISK	1.07 (0.21–5.48)	0.931
MODERATE RISK	4.49 (1.46–13.77)	0.009
HIGH RISK	9.34 (3–29.03)	<0.001
WMR		
Normal	1	-
Risky	1.60 (0.78–3.28)	0.202
WBC (×10^3^/µL)		
Normal	1	-
Risky	1.60 (0.88–2.93)	0.127
Neutrophil (×10^3^/µL)		
Normal	1	-
Risky	0.88 (0.46–1.68)	0.690
χ^2^ = 43.92; *p* < 0.001 p_Hosmer-Lemeshow Test_ = 0.715		

OR: Odds ratio; CI: confidence interval. The “very low risk” category was used as the reference category for T-MACS classification. “Normal” categories were used as reference groups for WMR, WBC, and neutrophil measurements. Cut-off values for WMR, WBC, and neutrophil count were determined using ROC curve analysis for 30-day MACE prediction. Values above the identified ROC-derived cut-off thresholds were categorized as “risky,” whereas values below these thresholds were categorized as “normal.”.

## Data Availability

The data that support the findings of this study are not publicly available due to patient confidentiality and institutional ethical restrictions, but are available from the corresponding author upon reasonable request and with appropriate ethical approvals.
